# Inflammatory activity evaluation in patients with axial spondyloarthritis using MRI relaxometry and mucosal-associated invariant T cells

**DOI:** 10.3389/fimmu.2024.1391280

**Published:** 2024-05-22

**Authors:** Shengsheng Yang, Yonghong Zheng, Xianyuan Chen, Mingui Lin, Xiaomin Dai, Fei Gao, Huangjing Chen, Mingping Ma, Shun Yu

**Affiliations:** ^1^ Shengli Clinical Medical College of Fujian Medical University, Fujian, Fuzhou, China; ^2^ Department of Radiology, Fujian Provincial Hospital, Fujian, Fuzhou, China; ^3^ Department of Radiology, Fuzhou Second Hospital, Fujian, Fuzhou, China; ^4^ Department of Rheumatism, Fujian Provincial Hospital, Fujian, Fuzhou, China

**Keywords:** axial spondyloarthritis, magnetic resonance, T1 mapping, mucosal-associated invariant T cells, combined-parameter model

## Abstract

**Background:**

Currently, there is a lack of an objective quantitative measure to comprehensively evaluate the inflammatory activity of axSpA, which poses certain challenges in accurately assessing the disease activity.

**Objective:**

To explore the value of combined-parameter models of sacroiliac joints (SIJs) MRI relaxometry and peripheral blood Mucosal-associated invariant T (MAIT) cells in evaluating the inflammatory activity of axial spondyloarthritis (axSpA).

**Methods:**

This retrospective clinical study included 88 axSpA patients (median age 31.0 (22.0, 41.8) years, 21.6% females) and 20 controls (median age 28.0 (20.5, 49.5) years, 40.0% females). The axSpA group was classified into active subgroup (n=50) and inactive subgroup (n=38) based on ASDAS-CRP. All participants underwent SIJs MRI examination including T1 and T2* mapping, and peripheral blood flow cytometry analysis of MAIT cells (defined as CD3^+^Vα7.2^+^CD161^+^) and their activation markers (CD69). The T1 and T2* values, as were the percentages of MAIT cells and CD69^+^MAIT cells were compared between different groups. Combined-parameter models were established using logistic regression, and ROC curves were employed to evaluate the diagnostic efficacy.

**Results:**

The T1 values of SIJs and %CD69^+^MAIT cells in the axSpA group and its subgroup were higher than the control group (p<0.05), while %MAIT cells were lower than the control group (p<0.05). The T1 values and %CD69^+^MAIT cells correlated positively, while %MAIT cells correlated negatively, with the ASDAS-CRP (r=0.555, 0.524, -0.357, p<0.001). Between the control and axSpA groups, and between the inactive and active subgroups, the combined-parameter model T1 mapping+%CD69^+^MAIT cells has the best efficacy (AUC=0.959, 0.879, sensibility=88.6, 70%, specificity=95.0, 94.7%, respectively).

**Conclusion:**

The combined-parameter model T1 mapping+%CD69^+^MAIT cells allows a more accurate evaluation of the level of inflammatory activity.

## Introduction

Axial Spondyloarthritis (axSpA) is a chronic inflammatory disease primarily affecting the sacroiliac joints (SIJs), clinically manifested as inflammatory low back pain and morning stiffness, which is often worsened at rest and relieved after exercise (1, 2). It triggers inflammatory changes in SIJs, insidiously progressing to structural changes, such as fat metaplasia, erosion, and ankylosis (3, 4). Although the mechanisms leading to the progression remain elusive, several studies highlighted the impact of persistent higher disease activity in facilitating structural damage (5, 6), which is intimately associated with poor prognosis (7). Therefore, early diagnosis and treatment of axSpA to reduce the inflammatory activity is pivotal in halting progressive disease progression, optimizing joint function and improving the quality of life (8–10).

Ankylosing Spondylitis Disease Activity Score (ASDAS), a composite score assessing disease activity in axSpA, combines objective and subjective indicators with varying weights (11). It shows relatively strong feasibility and sensitivity, along with better psychometric properties, making it the recommended preferred indicator in clinical practice (10, 12). Nevertheless, it mainly evaluates the symptoms of patients, making it inherently difficult to ensure the accuracy.

MRI is an important tool for the early diagnosis of axSpA. Subchondral bone marrow edema (BME) in the SIJs is a required finding to fulfill the Assessment of SpondyloArthritis International Society (ASAS) criterion of positive MRI findings as a part of the classification criteria for axSpA (1, 13, 14). Magnetic relaxometry technique, detecting subtle changes of water content of the tissue, can quantitatively assess the extent of BME (15, 16). Previous study showed that T1 mapping and T2* mapping values were effective quantitative indicators for clinical diagnosis, disease activity assessment and treatment monitoring in axSpA (17). However, due to the heterogeneity and the fluctuating nature of inflammatory activity in axSpA, relying solely on quantitative detection of BME is insufficient to evaluate the overall inflammatory activity level in axSpA patients (18, 19).

Mucosal-associated invariant T (MAIT) cells (defined as CD3^+^Vα7.2^+^CD161^+)^, as a unique subset of T lymphocytes, exhibit innate and acquired immune characteristics (20–22). The secretion of cytokines such as tumor necrosis factor and Interleukin-17 by MAIT cells has been recognized as an important role in the occurrence and progression of inflammation in axSpA, and the treatment strategies targeting these cytokines have been widely applied in clinical practice (22–24). Previous studies have shown the percentages of MAIT cells in the peripheral blood were reduced in patients with axSpA, and the CD69 expression on MAIT cells correlated with the severity of axSpA (25). However, there is limited research on the MAIT cells and CD69^+^MAIT cells in evaluating inflammatory activity in axSpA.

This study combines MRI relaxometry values (T1 values, T2* values) with MAIT cell-related parameters (%MAIT cells, %CD69^+^MAIT cells) to explore a more reliable and objective combined-parameter model that monitors the disease activity of axSpA.

## Materials and methods

This retrospective clinical study was approved by the institutional ethics committee (K2020-07-023), and the requirement for written informed consent was waived.

### Subjects

From September 2021 to June 2023, patients referred to the Rheumatology Department of our hospital and diagnosed with axSpA or mechanical lower back pain were collected in this study. According to the inclusion and exclusion criteria, 88 patients were included in the axSpA group, and 20 patients were included in the control group (**Supplementary Figure S1**).

The inclusion criteria in the axSpA group: (1) patients who met the 2019 ASAS criteria (13); (2) the clinical and MRI data are complete. The inclusion criteria in the control group: (1) patients who underwent SIJ MRI examination due to mechanical lower back pain; (2) absence of high signal intensity in the subchondral bone marrow of bilateral SIJs on the intermediate weighted imaging with fat suppression (IWI-FS) sequence; (3) not accord with the ASAS diagnostic criteria for axSpA; (4) the clinical and MRI data are complete. Exclusion criteria: (1) MRI contraindications; (2) poor image quality, presence of MRI artifacts; (3) with a history of autoimmune disease other than axSpA; (4) with tumors, trauma, infection or other lesions in the SIJs; (5) history of using hormones and immune drugs in recent 6 months; (6) hemolysis occurred during the flow cytometry assay.

### ASDAS-CRP score and grouping

This study utilized the ASDAS-CRP based on C-reactive protein (CRP) as the standard for assessing disease activity in axSpA patients. ASDAS-CRP is calculated based on the patient’s history of back pain, peripheral pain, duration of morning stiffness, overall pain assessment, and CRP over the past week (11). The ASDAS-CRP formula is as follows:


ASDAS−CRP=[0.121 × back pain + 0.058 × duration of morning stiffness + 0.110 × patient global assessment + 0.073 × peripheral pain and swelling + 0.579 × ln(CRP + 1)]

(CRP is in mg/L, using 2 if below the threshold or <2 in the high-sensitivity CRP test).

If ASDAS-CRP<1.3, the axSpA patients are classified as the inactive subgroup, and if ASDAS-CRP≥1.3, the axSpA patients are classified as the active subgroup (11). A rheumatologist with more than 15 years of work experience evaluated the ASDAS-CRP, accurately categorizing the axSpA patients into active and inactive subgroups.

### MRI examinations

All participants underwent the SIJ MRI scan using a 1.5T MRI scanner (MAGNETOM Aera, SIEMENS Healthcare, Erlangen, Germany) equipped with an 18-channel phased array torso coil. All participants were positioned in the supine position with the head entering first. The positioning line was placed at the level of bilateral anterior superior spines. All sequences were acquired by scanning in the oblique axis planes or oblique coronal planes (perpendicular or parallel to the axis of the sacral plane). The scanning sequences and parameters are shown in the **Table 1**.

**Table 1:** Acquisition parameters of MRI.

Imaging plane	T1WI	T2WI	T2WI-FS	IWI-FS	T1 mapping	T2* mapping
	Coronal oblique	Axial oblique	Axial oblique	Coronal oblique	Coronal oblique	Coronal oblique
TR/TE (ms)	582/7.4	3200/68	4000/57	3880/43	11/1.57	422/(4.18/11.32/ 18.46/25.60/32.74)
FOV (mm)	240	220	220	240	240	240
Slice thickness (mm)	3.0	4.0	4.0	3.0	3.0	3.0
Flip angle	150	150	150	150	5/27	60
No. of slice	18	20	20	18	18	18
Matrix	256×320	256×320	205×256	205×256	256×256	256×256
Voxel size (mm^3^)	0.8×0.8×3.0	0.7×0.7×4.0	0.9×0.9×4.0	0.9×0.9×3.0	0.5×0.5×3.0	0.9×0.9×3.0
Bandwidth (Hz/Px)	223	193	193	171	430	260
Parallel imaging	GRAPPA 2	GRAPPA 2	GRAPPA 2	GRAPPA 2	GRAPPA 2	GRAPPA 2
Fat suppression	NONE	NONE	SPAIR	SPAIR	NONE	NONE
Acquisition time (min:s)	1:41	1:04	2:00	2:04	1:50	3:46

FOV, field of view; IWI-FS, the intermediate weighted imaging with fat suppression; TR/TE, repetition time/echo time.

### Image analysis

T1 mapping and T2* mapping imaging were processed by Siemens syngo MRD13 image post-processing workstation, and the corresponding pseudo-color maps were generated. Two trained raters (7 and 15 years experience in musculoskeletal imaging) independently manually delineated regions of interest (ROIs) on the T1 mapping and T2* mapping pseudo-color maps, and were blinded to the clinical information of the participants. Referring to conventional MRI sequences, the sacroiliac joint (SIJ) was segmented into four subchondral areas (left ilium, left sacrum, right ilium, and right sacrum), with three regions of interest (ROIs) placed on each subchondral area. To guide the placement of the ROIs, BME was used as a reference for active inflammatory changes, with the raters identifying a hyperintense region on the IWI-FS sequence.

**Figure 1:**
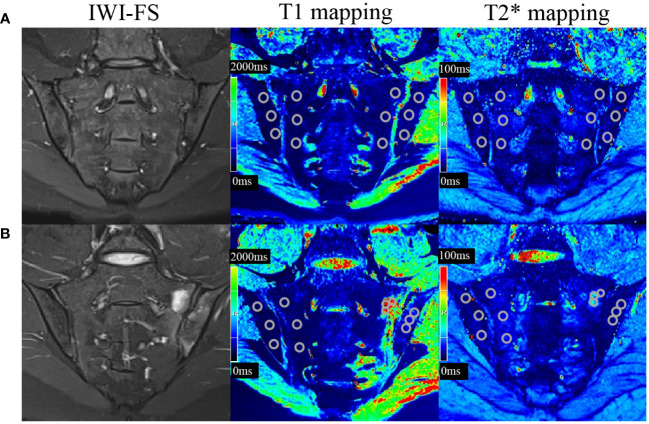
T1 and T2* mapping measurements in the sacroiliac joints without or with bone marrow edema (BME). Cases of regions of interest placement in the participant without BME **(A)** and with BME **(B)**.

The overall measurement principles were established as follows: if BME was detected in the subchondral bone, three non-overlapping ROIs (25–35 mm^2^) were respectively placed on areas of the T1 and T2* mapping corresponding with the highest signal intensity, which were adjusted according to the size of lesions. If BME was not observed, then three ROIs (25–35 mm^2^) were placed on the superior, middle, and inferior areas of the subchondral bone marrow (**Figure 1**). Bone cortex, sclerosis, blood vessels and the cystic area were avoided while placing the ROI. Through this process, 12 ROIs were obtained for T1 mapping or T2* mapping sequence for each participant. The mean T1 mapping or T2* mapping values for three ROIs within each subchondral area were calculated, and the maximum value among the four subchondral areas was considered the result. The average of the values assessed by two independent raters was used as the ultimate outcome.

### Flow cytometry assay

The percentages of MAIT cells and CD69^+^MAIT cells were detected by flow cytometry analysis: Fresh peripheral blood mononuclear cells (PBMCs) were isolated from heparinized blood by gradient centrifugation, washed with phosphate-buffered saline (PBS), and then resuspended and counted. The PBMCs were transferred to a flow cytometry tube and then were stained with the following combinations of monoclonal antibodies: 5ul FITC-anti-human-CD3^+^, 5ul phycoerythrin (PE)-anti-human-Vα7.2^+^, 20ul allophycocyanin (APC)-mouse anti-human-CD161^+^ and 5ul Peridinin-chlorophyll-protein complex-Cyanine 5 (Percp-Cy 5)-anti-human-CD69^+^ (Becton, Dickinson and Company, USA). Samples were incubated for 20 minutes at room temperature in the dark. Afterward, samples were washed with PBS and were resuspended, and counted again. The percentages of each cell population were analyzed using a flow cytometer (BD Accuri C6 PLUS). Lymphocytes were selected for analysis based on their forward and side scatter characteristics. The populations that were positively and negatively stained were determined using quadrant dot-plot analysis. MAIT cells were identified as CD3^+^Vα7.2^+^CD161^+^ cells, and CD69^+^ MAIT cells were identified as CD3^+^Vα7.2^+^CD161^+^CD69^+^ cells (**Supplementary Figure S2**).

### Statistical analysis

All data were analyzed using the Statistical Package for the Social Sciences 25.0 (SPSS, Inc., Chicago, IL, USA). Categorical variables are presented as frequencies. Shapiro-Wilk test was used to assessed the normality of continuous variables, which are expressed as mean ± standard deviations (SD) (normal distribution) or median (Q1, Q3) (non-normal distribution). The interobserver agreement of the T1 mapping and T2* mapping values measurements were analyzed using the interclass correlation coefficient (ICC) (< 0.40, poor; 0.40–0.59, fair; 0.60–0.74, good; ≥0.75, excellent). Spearman’s rho analyzed the correlation between ASDAS-CRP and T1 values, T2* values, %MAIT cells as well as %CD69^+^MAIT cells. The χ^2^ test or Fisher exact test was used to compare for categorical variables.The Mann-Whitney U test was to compare the difference of continuous variables between the control group and the axSpA group. The Kruskal-Wallis H test was used to compare the difference of continuous variables among three groups (control group, inactive subgroup and active subgroup). Bonferroni correction was used to adjust the p values for multiple comparisons. Logistic regression was used to construct combined-parameter models. The receiver operating characteristic (ROC) curves were used to analyze the diagnostic efficacy of the single-parameter and combined-parameter models. The area under the curve (AUC), optimal cut-off values, sensitivity, and specificity were calculated. For comparisons of pairwise AUCs, DeLong’s test was employed. The McNemar Test was used to compare the sensitivity and specificity of the models. A p-value<0.05 was indicative of statistical significance in all analyses.

## Results

### Demographic and clinical characteristics

A total of 88 patients were eventually included in the axSpA group and 20 individuals were included in the control group. According to ASDAS-CRP, 38 patients were classified as the inactive subgroup (ASDAS-CRP< 1.3), and 50 patients were classified as the active subgroup (ASDAS-CRP≥ 1.3) (**Table 2**). There were no statistically differences between the axSpA group and the control group in terms of age[(31.0 (22.0, 41.8) years in the axSpA group vs. 28.0 (20.5, 49.5) years in the control group, p=0.785)] and gender [69 (21.6%) female in axSpA group vs. 12 (40.0%) female in control, p=0.086].

**Table 2:** Demographic and clinical characteristics.

	control group (n=20)	axSpA group (n=88)	axSpA group (n=88)	
			inactive subgroup (n=38)	active subgroup (n=50)
Age (y)^‡^	28.0(20.5, 49.5)	31.0 (22.0, 41.8)	28.5 (22.8, 40.0)	31.5 (22.0, 46.0)
Sex^‡‡^				
Male	12 (60.0%)	69 (78.4%)	30 (78.9%)	39 (78.0%)
Female	8 (40.0%)	19 (21.6%)	8 (21.1%)	11 (22.0%)
HLA-B27^‡‡^				
+	0 (0.0%)	80 (90.9%)	36 (94.7%)	44 (88.0%)
−	20 (100.0%)	8 (9.1%)	2 (5.3%)	6 (12.0%)
CRP (mg/L)^‡^	2.00 (2.00, 2.00)	2.39 (2.00, 11.78)	2.00 (2.00, 2.00)	10.20 (3.39, 28.98)
ASDAS-CRP^‡^	—	1.65 (0.73, 2.70)	0.60 (0.60, 1.10)	2.65 (2.00, 3.20)
BME^‡‡^	0 (0.0%)	73(83.0%)	26(68.4%)	47(94.0%)

ASDAS-CRP, Ankylosing Spondylitis Disease Activity Score–C-reactive protein; CRP, C-reactive protein; HLA-B27, human leukocyte antigen-B27.

^‡^ median (Q1, Q3).

^‡‡^ n (%).

### Analyses of interobserver agreement

There was excellent interobserver agreement between the two raters for T1 values and T2* values measurements (ICC: 0.922, CI:0.887-0.946 and ICC:0.867, CI:0.812-0.907, respectively).

### Correlations of the ASDAS-CRP with MRI relaxometry values and MAIT cells-related parameters

**Figure 2:**
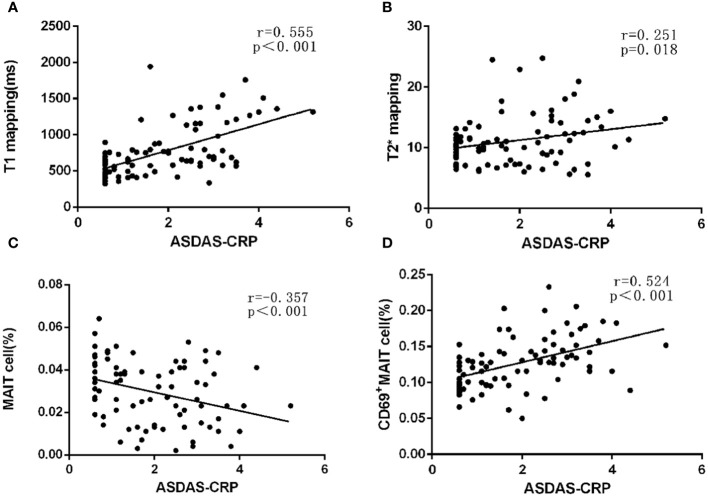
Correlations of the ASDAS-CRP with MRI relaxometry values and MAIT cells-related parameters. **(A) **The correlations of the ASDAS-CRP with T1 values. **(B) **The correlations of the ASDAS-CRP with T2* values. **(C)** The correlations of the ASDAS-CRP with %MAIT cells. **(D)** The correlations of the ASDAS-CRP with %CD69^+^MAIT cells.

Spearman’s rho analysis revealed that the T1 values and %CD69^+^MAIT cells in the axSpA groups exhibited positive correlations with the ASDAS-CRP. The correlation coefficients (r) were 0.555 and 0.524 (all p<0.001). On the other hand, %MAIT cells in the axSpA group showed a negative correlation with the ASDAS-CRP (r=-0.357, p<0.001). T2* values exhibited no correlations with the ASDAS-CRP (r=0.251, p=0.018) (**Figure 2**).

### The intergroup comparison of T1 values and T2* values

**Figure 3:**
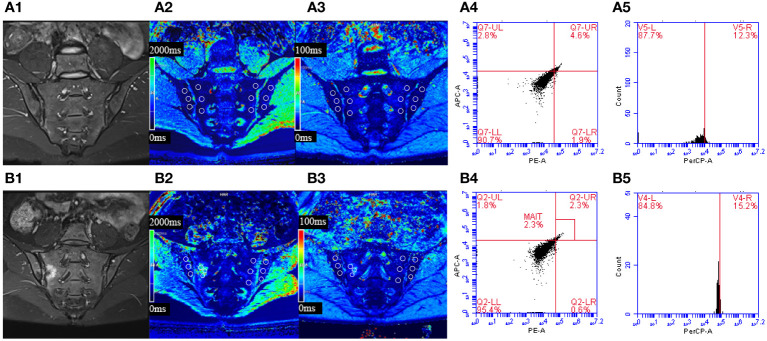
**(A)** Male, 23 years old, HLA-B27: +, CRP:<0.715mg/L, ASDAS-CRP: 0.6, belonged to the inactive group. **(B)** Female, 30 years old, HLA-B27: +, CRP: 88.3mg/L, ASDAS-CRP: 5.2, belonged to the active group. **(A1)** The coronal plane of IWI-FS showed no obvious high signal intensity in the subchondral bone of bilateral sacroiliac joints. **(A2, A3)** Pseudo-color T1 and T2* mapping imaging demonstrates the T1 and T2* value were 653.73 ms and 9.3 ms, respectively. **(A4, A5)** The percentages of MAIT cells and CD69^+^MAIT cells were 4.6% and 12.3%, respectively. **(B1)** The coronal plane of IWI-FS showed obvious high signal intensity in the subchondral bone of right sacroiliac joints, and the corresponding articular surfaces are rough. **(B2, B3)** Pseudo-color T1 and T2* mapping imaging demonstrates the T1 and T2* value were 1317.0 ms and 14.8 ms, respectively. **(B4, B5)** The percentages of MAIT cells and CD69^+^MAIT cells are 2.3% and 15.2%, respectively.

T1 values in the axSpA group, inactive subgroup, active subgroup were higher than the control group (p<0.001, p=0.005, p<0.001), and the active subgroup had higher T1 values than the inactive subgroup (p<0.001). The T2* values of the axSpA group and the active subgroup were higher than the control group (p=0.038, p=0.031), but there were no statistical differences between the inactive subgroup and the control group (p=0.836), as well between the inactive subgroup and the active subgroup (p=0.232) (**Tables 3**, **4**, **Figures 3A1-A3, B1-B3**).

**Table 3:** Intergroup comparison of MRI relaxation values and MAIT cells-related parameters between control group and axSpA group.

	control group (n=20)	axSpA group (n=88)	*P*†
MAIT cells (%)	5.15 (4.53, 6.25)	3.20 (1.73, 4.18)	<0.001
CD69^+^MAIT cells (%)	7.55 (5.25, 9.43)	12.60 (9.85, 14.38)	<0.001
T1 mapping (ms)	393.98 (350.33, 434.70)	656.33 (524.86, 880.22)	<0.001
T2* mapping (ms)	9.18 (7.61, 10.03)	10.33 (8.40, 13.16)	0.038

† Mann-Whitney U test.

**Table 4:** Intergroup comparison of MRI relaxation values and MAIT cells-related parameters among the control, inactive, and active groups.

	control group (n=20)	axSpA group		*P*†	*P*_a_‡	*P*_b_‡	*P*_c_‡
		inactive subgroup (n=38)	active subgroup (n=50)				
MAIT cells (%)	5.15 (4.53, 6.25)	3.80 (2.93, 4.60)	2.30 (1.28, 3.83)	<0.001	0.043	<0.001	0.001
CD69^+^MAITcells (%)	7.55 (5.25, 9.43)	10.10 (9.28, 12.40)	13.90 (11.60, 16.78)	<0.001	0.016	<0.001	<0.001
T1 mapping (ms)	393.98 (350.33, 434.70)	568.65 (419.71, 655.72)	790.15 (630.75, 1228.10)	<0.001	0.005	<0.001	<0.001
T2* mapping (ms)	9.18 (7.61, 10.03)	9.83 (8.40, 11.48)	11.30 (7.84, 15.12)	0.024	0.836	0.031	0.232

†Kruskal-Wallis H test; ‡Bonferroni correction.

a. Derived from the comparison between the control group and inactive group.

b. Derived from the comparison between the control group and active group.

c. Derived from the comparison between the inactive group and active group.

### The intergroup comparison of %MAIT cells and %CD69^+^MAIT cells

The percentages of MAIT cells in the axSpA group, inactive subgroup and active subgroup were lower than the control group (p<0.001, p=0.043, p<0.001), and the active subgroups had lower %MAIT cells than the inactive subgroups (p=0.001).Conversely, %CD69^+^MAIT cells in the axSpA group, active subgroup, and inactive subgroup were higher compared to the control group (p<0.001, p=0.016, p<0.001). Moreover, %CD69^+^MAIT cells were higher in the active subgroup compared to the inactive subgroup (p<0.001) (**Tables 3**, **4**, **Figures 3A4, A5, B4, B5**).

### Comparison of single-parameter and combined-parameter models

**Figure 4:**
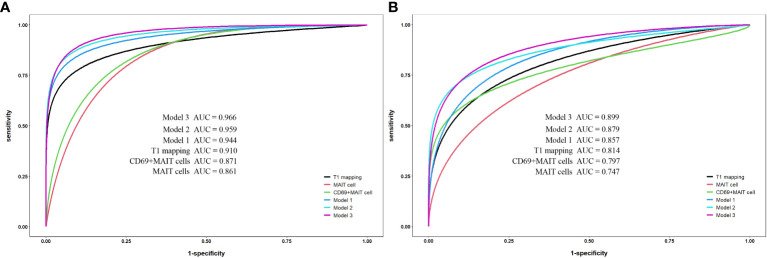
Receiver operating characteristic curve analysis for single-parameter and combined-parameter models in the diagnosis and inflammation activity assessment of axSpA. **(A)** Between the control group and axSpA group. **(B)** Between the inactive group and active group. Model 1:T1 mapping+%MAIT cells; Model 2:T1 mapping +%CD69^+^MAIT cells; Model 3:T1 mpping+%MAIT cells+%CD69^+^MAIT cells.

For the single-parameter models, T1 mapping exhibited the highest AUC values of the three single-parameters between the control and axSpA groups, as well as between the inactive and active subgroups. For the bivariate combined-parameter models, the AUC values all higher than the single-parameter models, and the AUC values of model 2 (T1 mapping+%CD69^+^MAIT cells) were higher than model 1 (T1 mapping+%MAIT cells). For the trivariate combined-parameter model 3 (T1 mapping+%MAIT cells+%CD69^+^MAIT cells), it yielded the highest AUC values (**Table 5**, **Figure 4**).

**Table 5:** Diagnostic performance of single-parameter and combined-parameter models in diagnosing and distinguishing different activity.

	Model	AUC	95%CI	Sensitivity	Specificity	Cut-off	P
**control group** **vs.** **axSpA group**	MAIT cells (%)	0.861	0.764-0.959	81.8	80.0	4.50	<0.001
	CD69^+^MAIT cells (%)	0.871	0.783-0.959	78.4	85.0	9.60	<0.001
	T1 mapping (ms)	0.910	0.856-0.964	81.8	95.0	476.77	<0.001
	Model 1^a^	0.944	0.902-0.987	88.6	90.0	0.67	<0.001
	Model 2^b^	0.959	0.926-0.992	88.6	95.0	0.69	<0.001
	Model 3^c^	0.966	0.934-0.999	94.3	90.3	0.64	<0.001
**inactive group** **vs.** **active group**	MAIT cells (%)	0.747	0.645-0.849	58.0	86.8	2.60	<0.001
	CD69^+^MAIT cells (%)	0.797	0.702-0.891	64.0	89.5	12.90	<0.001
	T1 mapping (ms)	0.814	0.727-0.901	60.0	89.5	748.87	<0.001
	Model 1^a^	0.857	0.781-0.932	64.0	92.1	0.66	<0.001
	Model 2^b^	0.879	0.808-0.950	70.0	94.7	0.64	<0.001
	Model 3^c^	0.899	0.837-0.961	86.0	81.6	0.49	<0.001

AUC, area under the curve; CI, confidence intervals; MAIT cells, Mucosal-associated invariant T cells.

a. Model 1: T1 mapping+%MAIT cells.

b. Model 2: T1 mapping+%CD69^+^MAIT cells.

c. Model 3: T1 mapping+%MAIT cells+%CD69^+^MAIT cells.

According to DeLong’s test (**Table 6**), there were no statistical differences between the AUC values of T1 mapping with %MAIT cells, %CD69^+^MAIT cells and model 1 (p=0.0657-0.8535). And the AUC of model 2 and model 3 exhibited statistical difference compared to T1 mapping (p=0.0146-0.0471), but the model 2 exhibited no statistical difference compared to model 3 (p=0.3486-0.3904). The result of McNemar test suggest that the model 3 had better sensitivity than model 2 between the control group and axSpA group (p=0.0253), as well as between in the inactive subgroup and active subgroup (p=0.0047), but the specificity of model 3 was lower than model 2, especially between the inactive subgroup and active subgroup (P=0.0455) (**Supplementary Table S1**).

**Table 6:** DeLong’s test for the single-parameter and combined-parameter models.

Model	dAUC	SE	CI	Z	P
control group vs. axSpA group					
T1 mapping - %MAIT cells	0.049	0.052	-0.052 - 0.150	0.951	0.3418
T1 mapping - %CD69^+^MAIT cells	0.039	0.055	-0.068-0.146	0.717	0.4735
%MAIT cells - %CD69^+^MAIT cells	-0.010	0.054	-0.115-0.096	-0.185	0.8535
T1 mapping - Model 1^a^	-0.034	0.019	-0.070-0.002	-1.841	0.0657
T1 mapping - Model 2^b^	-0.049	0.022	-0.093–0.005	-2.171	0.0299
T1 mapping - Model 3^c^	-0.056	0.023	-0.101-0.011	-2.443	0.0146
Model 1^a^ - Model 2^b^	-0.015	0.017	-0.047-0.018	-0.888	0.3746
Model 1^a^ - Model 3^c^	-0.022	0.012	-0.046-0.002	-1.795	0.0726
Model 2^b^ - Model 3^c^	-0.007	0.008	-0.024-0.009	-0.859	0.3904
inactive group vs. active group					
T1 mapping - %MAIT cells	0.067	0.066	-0.062-0.195	1.016	0.3098
T1 mapping - %D69^+^MAIT cells	0.017	0.063	-0.107-0.141	0.266	0.7905
%MAIT cells - %CD69^+^MAIT cells	-0.050	0.070	-0.082-0.187	0.712	0.4766
T1 mapping - Model 1^a^	-0.043	0.033	-0.0212-0.108	-1.313	0.1890
T1 mapping - Model 2^b^	-0.065	0.033	-0.130-0.001	-1.931	0.0471
T1 mapping - Model 3^c^	-0.085	0.038	-0.159-0.012	-2.269	0.0232
Model 1^a^ - Model 2^b^	-0.022	0.038	-0.097-0.053	-0.576	0.5649
Model 1^a^ - Model 3^c^	-0.042	0.025	-0.091-0.007	-1.680	0.0930
Model 2^b^ - Model 3^c^	-0.020	0.021	-0.062-0.022	-0.937	0.3486

CI, confdence intervals; dAUC, difference between AUCs; MAIT cells, Mucosal-associated invariant T cells; SE, standard error.

a: Model 1:T1 mapping+%MAIT cells.

b: Model 2: T1 mapping+%CD69^+^MAIT cells.

c: Model 3:T1 mapping+%MAIT cells+%CD69^+^MAIT cells.

## Discussion

This study assessed the potential of combining MRI relaxometry values and MAIT cell-related parameters for quantitative assessment of the inflammatory activity in axSpA patients. The results suggested that T1 values, %MAIT cells and %CD69^+^MAIT can be used to evaluate the inflammatory activity of axSpA. And the combined-parameter T1 mapping+%CD69^+^MAIT cells is more conducive to improving the accuracy of discriminating the status of disease activity of axSpA.

T1 mapping is a quantitative MRI technique used to quantify the T1 relaxation time of tissues, and the signal depends on intracellular and extracellular factors (26). Therefore, histological characteristics can be quantitatively evaluated using T1 mapping images. Studies have indicated that T1 mapping can detect crucial pathological processes associated with edema, protein deposition, and other substances causing changes in T1 relaxation time, such as lipids or iron (hemorrhage, iron overload) (27). This study showed that the T1 values in the inactive subgroup were higher than those in the control group, suggesting even in the inactive subgroup, the water content in the subchondral bone marrow of the SIJs is higher than that in the control group. This may suggest that the inactive phase of axSpA is a subclinical or chronic disease process, where patients may experience ongoing inflammation or other pathological changes in the SIJs, even if they are currently not manifesting active symptoms of axSpA. Therefore, these results also emphasize the importance of regular monitoring and comprehensive management of the inactive phase of axSpA.

T2* mapping, utilizing the unique features including speed of imaging and high image resolution, and the ability to carry out isotropic three-dimensional cartilage evaluation, has been widely used to assess the health condition of joint cartilage (28). Several studies have suggested that T2* mapping can provide quantitative information about the integrity of collagen fibers and changes in bound water, making it a potential method for the early assessment of osteoarthritis (29, 30). Due to the intricate anatomy of the SIJs and the thinness of their cartilage, impacting T2* imaging accuracy (31), our study differs by placing the ROIs in the subchondral bone. Some studies indicated T2* mapping may not be sensitive enough to detect small-scale pathological changes or early lesions (32). Meanwhile, the presence of sensitivity artifacts caused by the lack of 180 refocusing pulses in the T2*mapping can significantly affect the T2* mapping evaluation (32). This may explain why there were statistical differences in T2* values between the axSpA group and control groups in our study, but there were no statistical differences between the control group and inactive, as well as between the inactive subgroup and active subgroup. Therefore, compared to the more sensitive T1 mapping technology, T2* mapping serves only as a supplementary method for assessing the activity levels in axSpA.

MAIT cells are essential components of mucosal immunity, constituting 1%-10% of the total T cell population in the body (33). In recent years, MAIT cells have garnered increasing attention for their functions and impact on autoimmune diseases. Previous research indicated that the percentage of MAIT cells is reduced in patients with Systemic Lupus Erythematosus (SLE), conversely, in SLE patients with active disease, there is an increase in the activation state of MAIT cells (34). Similarly, MAIT cells were observed to migrate from the peripheral blood to inflamed mucosal tissues in patients with Crohn’s Disease (CD) and Ulcerative Colitis (UC), releasing inflammatory cytokines such as IL-17A and IL-22. Additionally, the expression of CD69^+^MAIT cells correlates with disease activity (34). These findings collectively suggested the involvement of MAIT cells in autoimmune inflammatory responses.

In this study, the percentages of MAIT cells in the peripheral blood of the axSpA group were significantly lower than that in the control group. This decrease may be related to the tendency of MAIT cells to engage in inflammatory responses within SIJs. Additionally, the percentages of MAIT cells in the active subgroup were also significantly lower than that in the inactive subgroup, indicating a correlation between MAIT cells and the induction of active inflammation. In contrast, axSpA patients showed elevated percentages of CD69^+^MAIT cells, which were significantly and positively correlated with ASDAS, indicating a relationship with disease activity to some extent. Studying the characteristics and functions of MAIT cells can help understand immune responses and the mechanisms of disease occurrence in axSpA, and will provide new insights for the development of related treatment strategies.

Previous studies have only examined the assessment of axSpA activity from a single perspective. This study combined the MRI relaxometry values and MAIT cell-related parameters, significantly improving the accuracy for assessment of the activity of axSpA. Especially when T1 mapping is combined with %CD69^+^MAIT cells, its AUC, sensitivity, and specificity are significantly improved compared to T1 mapping alone, suggesting these two indicators may have a complementary effect in the evaluation of axSpA activity. T1 mapping can directly show the morphology and distribution of BME and can quantitatively evaluate BME, while CD69^+^MAIT cells in the peripheral blood can quantitatively assess the inflammatory activity in axSpA patients. AxSpA is a heterogeneous disease, and the presence of BME in the SIJs can vary among individuals. Some patients may exhibit high ASDAS even without BME in the SIJs, which may suggest there exists a potential lag or fluctuation in BME during active inflammation (35). In such cases, monitoring the percentages of CD69^+^MAIT cells in peripheral blood can provide valuable complementary information. However, it’s essential to note that there is a lack of specificity in evaluating axSpA based on the expression of MAIT cells, as they are correlated with the activity of various immune diseases. Since this study excluded other rheumatic diseases, it might overestimate the sensitivity and specificity of MAIT cells. The combined-parameter model T1 mapping+%CD69^+^MAIT cells allows for a more holistic evaluation assessment of axSpA disease activity.

We acknowledge several limitations. First, the small-sample study without subdividing the activity subgroups may result in our conclusions being somewhat inconclusive, though setting strict inclusion and exclusion criteria can ensure the similar characteristics and conditions of the participants, reduce the impact of individual differences and ensure the consistency and comparability of results. Further validation is needed through larger sample sizes, multicenter, and randomized controlled trials. Second, the absence of observed gender differences is likely due to type 2 error (i.e. false negative due to small study set), which can be further verified by increasing the sample size. Third, the lack of histological validation on the SIJ lesions is a thorny issue, warranting further research on the correlation between histopathological characteristics and radiological features in sacroiliitis. In addition to BME, active inflammation in the SIJs of axSpA can also manifest as synovitis, enthesitis, etc. This study primarily focuses on the relationship between BME and activity levels, overlooking the investigation of other signs of active inflammation. Finally, this study did not evaluate the efficacy of quantitative parameters in monitoring treatment outcomes, which will be the focus of our future research.

In conclusion, T1 mapping values, %MAIT cells and %CD69^+^MAIT cells offer quantifiable indicators for inflammatory activity assessment of axSpA. Furthermore, the combined-parameter model T1 mapping+%CD69^+^MAIT cells enhances the efficacy in evaluating the disease activity in axSpA.

## Data availability statement

The raw data supporting the conclusions of this article will be made available by the authors, without undue reservation.

## Ethics statement

The studies involving humans were approved by The Ethics Committee of Fujian Province Hospital. The studies were conducted in accordance with the local legislation and institutional requirements. The ethics committee/institutional review board waived the requirement of written informed consent for participation from the participants or the participants’ legal guardians/next of kin because this retrospective clinical study was approved by the institutional ethics committee, and the requirement for written informed consent was waived.

## Author contributions

SSY: Conceptualization, Data curation, Methodology, Writing – original draft, Writing – review & editing, Formal Analysis, Investigation, Resources. YHZ: Data curation, Writing – original draft, Writing – review & editing, Formal Analysis, Investigation, Project administration. XYC: Writing – original draft, Writing – review & editing, Data curation, Formal Analysis, Investigation, Project administration, Validation. MGL: Conceptualization, Resources, Investigation, Software, Visualization, Writing – review & editing. XMD: Conceptualization, Investigation, Resources, Software, Visualization, Writing – review & editing. FG: Formal Analysis, Resources, Supervision, Validation, Writing – review & editing. HC: Formal Analysis, Resources, Supervision, Validation, Writing – review & editing. MPM: Formal Analysis, Resources, Supervision, Validation, Writing – review & editing. SY: Conceptualization, Formal Analysis, Funding acquisition, Methodology, Project administration, Supervision, Validation, Writing – review & editing.
