# Supplementary material for: Inflammatory activity evaluation in patients with axial spondyloarthritis using MRI relaxometry and mucosal-associated invariant T cells
Source: Front Immunol. 2024 May 22;15:1391280. doi: 10.3389/fimmu.2024.1391280 (PMC11150633; doi:10.3389/fimmu.2024.1391280)
Supplement: Supplementary file 1 [file DataSheet_1.docx]

**Table S1 Comparison of the sensitivity and specificity of model 2 and model 3**

|  | Index | Method 1 | Method 2 | Statistic | P |
| --- | --- | --- | --- | --- | --- |
| **control group vs. axSpA group** |  |  |  |  |  |
| Model 2 - Model 3 | sensitivity | 88.6% | 94.3% | 5 | 0.0253 |
| Model 2 - Model 3 | specificity | 95.0% | 90.0% | 1 | 0.3173 |
| **inactive subgroup vs. active group** |  |  |  |  |  |
| Model 2 - Model 3 | sensitivity | 70.0% | 86.0% | 8 | 0.0047 |
| Model 2 - Model 3 | specificity | 94.7% | 81.6% | 4 | 0.0455 |


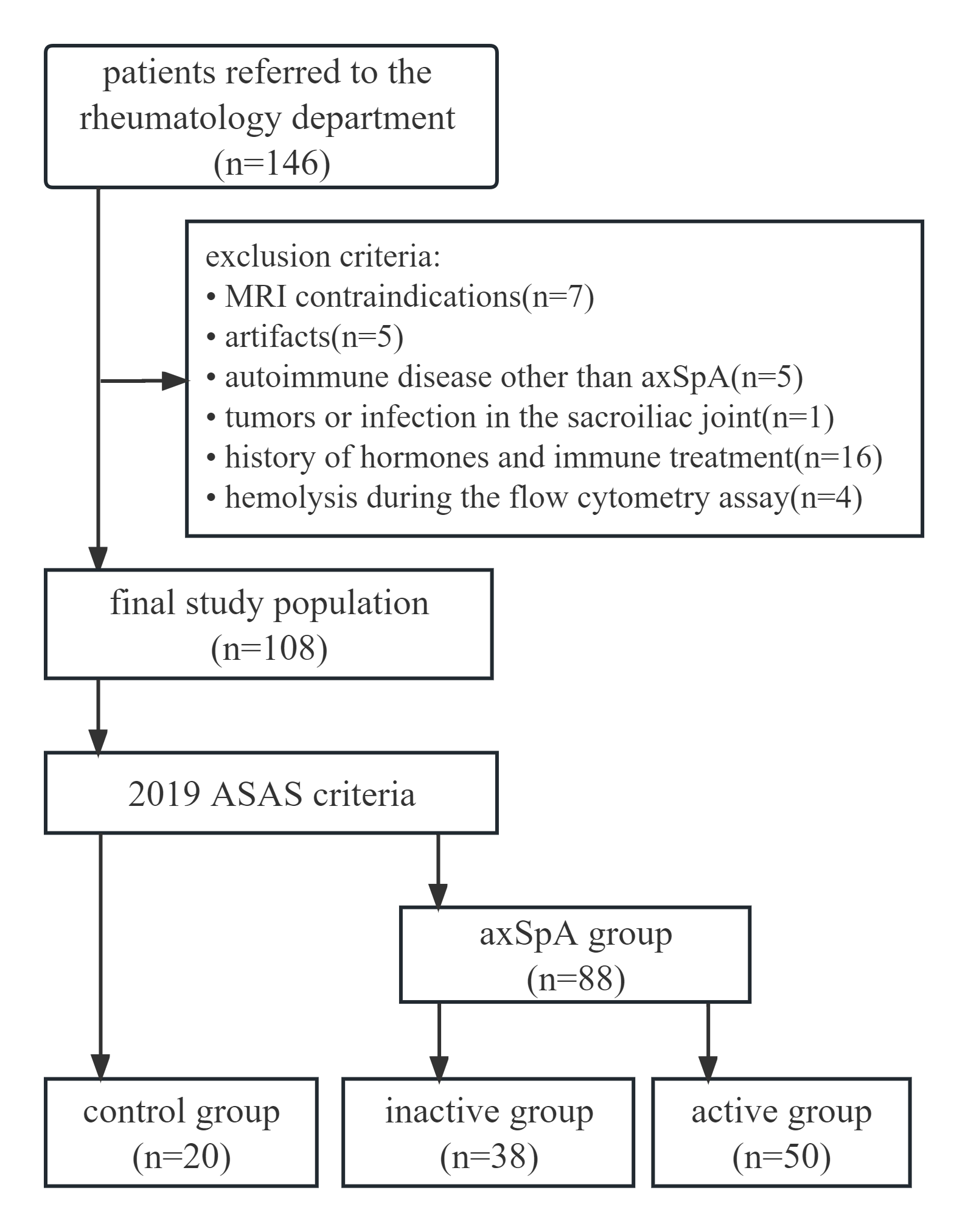


FIGURE S1: Flowchart of the study


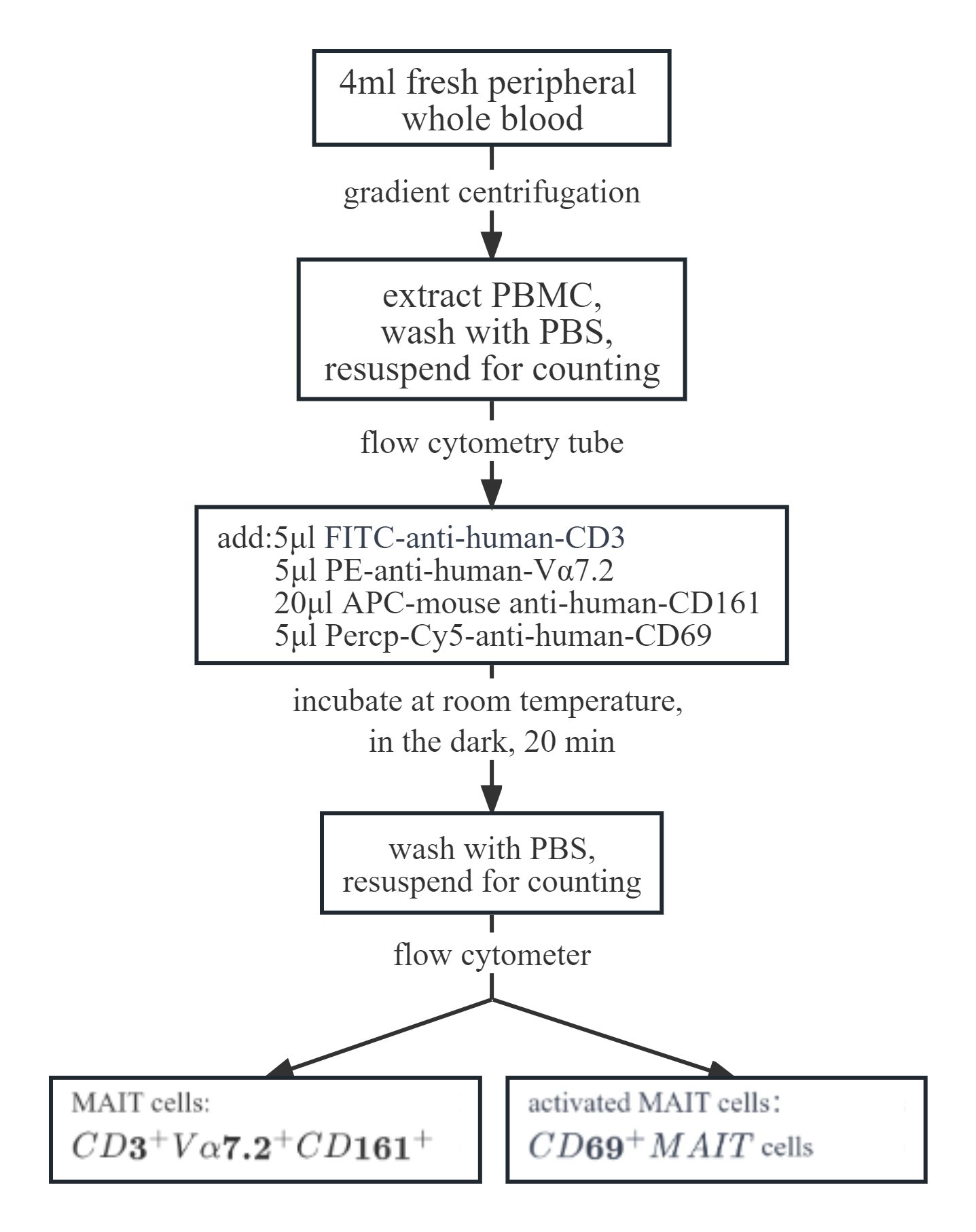
FIGURE S2: Flowchart of flow cytometry analysis
